# Exploring the paradox of Muslim advantage in undernutrition among under-5 children in India: a decomposition analysis

**DOI:** 10.1186/s12887-023-04345-y

**Published:** 2023-10-16

**Authors:** Shreya Banerjee, Shirisha P.

**Affiliations:** 1 Centre for the Study of Regional Development, Jawaharlal Nehru University, New Delhi, 110067 India; 2 Department of Humanities and Social Sciences, Indian Institute of Technology Madras, Chennai, 600036 Tamil Nadu India

**Keywords:** Undernutrition, Child health, Caste inequalities, Socioeconomic gradient, Religious disparity, Determinants, Decomposition

## Abstract

**Background:**

While there is a substantial body of research on inequalities in child nutrition along the axes of gender and socioeconomic gradient, the socio-religious differences in health and nutrition outcomes remain grossly understudied. The handful of studies on the socio-religious differential in child health outcomes has found a Muslim advantage in chances of survival and nutritional status over Hindus despite their comparatively lower socioeconomic status, which undeniably warrants investigating the pathways through which this paradoxical Muslim advantage manifests.

**Methods:**

Using data from the National Family Health Survey, 2015-16, we quantify the inter-group differentials in child undernutrition (stunting, wasting, and underweight) between Muslims and caste-disaggregated Hindus. We further decompose the gap to delineate its major contributory factors by employing Fairlie’s decomposition method.

**Results:**

The analysis revealed that, compared to the Hindus as an aggregated group, Muslims have a higher rate of stunting and lower rates of wasting and being underweight. However, the differences get altered when we disaggregate the Hindus into high and low castes. Muslims have a lower prevalence of all three measures of undernutrition than the low-caste Hindus and a higher prevalence of stunting and underweight than the high-caste Hindus, consistent with their levels of socioeconomic status. However, the prevalence of wasting among Muslim children is lower than among high-caste Hindus. This nutritional advantage is paradoxical because Muslims’ relatively poorer socioeconomic status compared to high-caste Hindus should have disadvantaged them. In the decomposition analysis, the Muslim advantage over the low-caste Hindus could only be partially attributed to the former’s better economic status and access to sanitation. Moreover, the poor performance of Muslim children compared to the high-caste Hindus in stunting and underweight could mainly be explained by the religious differentials in birth order, mother’s education, and wealth index. However, Muslim children’s comparatively better performance in wasting than the high-caste Hindus remained a puzzle.

**Conclusion:**

The Muslim advantage over high-caste Hindus in wasting and low-caste Hindus in all the indicators of undernutrition may have been rendered by certain ‘unobserved’ behavioural and cultural differences. However, further exploration is needed to make a definitive claim in this respect.

**Supplementary Information:**

The online version contains supplementary material available at 10.1186/s12887-023-04345-y.

## Background

‘Undernutrition’ and ‘overnutrition’ are the two facets of malnutrition that broadly refer to deficiencies, excesses, or imbalances in nutrients [1]. The effect of malnutrition as a potentiating factor for under-5 child deaths has often remained unrecognized [2–4]. As per an epidemiological study on 53 developing countries [3], India had the highest total Population Attributable Risk (PAR) to malnutrition (67%), and alarmingly, almost three-quarters of the total PAR was accounted for by mild-to-moderate malnutrition. Malnourished children, especially those suffering from severe acute malnutrition (SAM), are at a higher risk of death due to increased infectious diseases such as diarrhea, pneumonia, and malaria [1, 4].

Recent estimates show that while stunting has reduced globally, more than half of the stunted children and one-third of those wasted live in the South-Asian region [5]. Despite the implementation of the Integrated Child Development Scheme (ICDS), launched way back in 1975 to address the nutritional needs of children in India, the program has evidently not been a complete success in yielding positive outcomes. According to NFHS-5 (2019-20), the prevalence of undernutrition has either remained the same or worsened at the national level compared to the previous rounds, with only a handful of states showing a declining trend. This is mainly due to the deficit in effective coverage among the intended beneficiaries and poor quality of service delivery [6]. The global hunger Index, calculated based on overall undernourishment of the population, child stunting, child wasting, and child mortality, ranks India at the 94th position out of 107 countries, worse than its economically poorer neighboring countries such as Sri Lanka, Nepal, Bangladesh, Myanmar and Pakistan[7].

Furthermore, the disparities between the social strata and wealth quintiles have also widened or remained constant at best, rarely showing any shrinkage [8–10]. Several studies have previously examined the socioeconomic gradient of undernutrition and found it concentrated in households with poor socioeconomic status [11–14]. Also, numerous studies have observed gender disparities in malnutrition and other health indicators, with girls being disadvantaged due to widespread son preference among Indian households [15–19]. For example, Jayachandran & Kuziemko (2015) have advanced evidence suggesting the systematic neglect of female infants by being breastfed for a shorter duration than male infants [20]. While a large body of research supports the evidence that inequalities in nutrition status exist along the lines of gender and socioeconomic status, what remains understudied are the religious differences in health and nutrition outcomes.

There is a narrow body of research on the effect of religion on child survival in South Asian and African contexts [21–25]. The limited studies on the association between religion and health in India highlight a paradox wherein Muslims and Christians outperform Hindus in child health outcomes, despite Hindu advantages in socioeconomic status, employment, and education. As reported in one of the studies [24] conducted in India, Muslims outperformed Hindus by 2.31% points when considering the average child mortality rate between 1960 and 2006. Further, Muslim females benefit from this because there is less gender discrimination in the distribution of resources within Muslim households than in their Hindu counterparts [24–26]. In contrast, Menon and McQueeney (2015) discovered that Christian infants in India, particularly female newborns, exhibit an advantage in anthropometric outcomes over Hindu and Muslim infants [27]. Few other studies on the socio-religious differential in child health outcomes have found that Muslim children have reduced mortality and greater height-for-age compared to Hindu children [8, 24, 28]. Also, significant differences among the religious groups in women’s education and health status, personal health and hygiene, and access to medical care have been documented [27].

The Islamic conquests in India between the 12th and 16th centuries mainly led to the spread of Islam through the religious conversion of the indigenous population rather than the settlement of ‘foreigners’ [29]. This implies that Indian Muslims are genetically similar to the country’s Hindus. Moreover, historically, the position of Indian Muslims has been inferior to that of the Hindus in the socioeconomic gradient, as is evident from the following facts presented by the Post-Sachar Evaluation Committee report (2014) [30]. Muslims lag behind Hindus in educational attainment, with only 6% of the former completing graduation or a higher level of education and the highest dropout in secondary and higher secondary education. These inequalities in education reflect in the quality of employment among Muslims, who have a lower share of households earning a livelihood from regular wage employment and significantly less representation in government sector jobs. Labor force participation is the least among Muslim women (13.3%) compared to 21.25% among Hindu women [31]. Access to maternal and child health services has also been relatively lower among Muslim women. The rate of institutional deliveries among Muslims is 69% compared to 80.8% among Hindus, and the rate of antenatal care-seeking from skilled health providers is 77% compared to 79.3% among Hindus. The Hindu-Muslim differential in total fertility rate has reduced, but it still is marginally higher among Muslims (2.61%) than Hindus (2.13%). Similarly, the use of modern contraception among Muslims is 37.9% against 48.8% among Hindus [32].

The under-5 survival advantage among Muslim children is 17% higher when compared to Hindus [24]. This advantage comes as a surprise when contrasted with the findings of Caldwell (1986), observing higher child mortality rates in predominantly Muslim countries [33]. The observed advantage of Muslim children in survival and nutritional indicators is, therefore, all the more perplexing due to their inferior socioeconomic attributes coupled with the absence of any genetic dissimilarities compared to the Hindus.

However, at this point, it is essential to note the intra-group differences among the Hindus, who are segregated by several caste groups. The caste system practiced for ages is still deeply rooted in Indian society, particularly among the Hindus. Each caste is assigned a social status, where the upper castes have enjoyed better social status, and the lower castes (Scheduled Castes (SCs) and Scheduled Tribes (STs)) have often been at a disadvantage with regard to their socio-cultural rights. The caste system became a tool of oppression resulting in social disabilities and hindrances similar to the systems where inequity operates [34]. Thus, it would be a mistake to consider the Hindus as a homogenous group. Studies that have found Muslim children faring better than Hindu children in some of the nutritional indicators have also found that the differentials are more pronounced when Muslims are compared to Hindu lower castes (SCs and STs) [8, 28].

Given this backdrop, the present study aims to (i) quantify the inter-group differentials in child undernutrition (stunting, wasting, and underweight) between Muslims and caste-disaggregated Hindus and (ii) decompose the socio-religious differentials in the three indicators of child undernutrition into its major contributory factors.

There are two hypotheses that can explain the relationship between religion and child health. One is the Selectivity Hypothesis which suggests that the religious differentials can be attributed to the differences in access to resources and variations in living standards [22]. The other is the Particularized Theology Hypothesis, which suggests that the adoption or abstinence of a certain healthy lifestyle or health-damaging behavior, based on the doctrinal teachings, influences the child’s health and survival [35]. In this study, we have attempted to explore which of these hypotheses is more supported, if at all, by decomposing the differentials in nutritional outcomes between the communities into its major contributory factors.

## Methods

### Data

The present study draws evidence from the National Family Health Survey, 2015-16 (NFHS-4). The NFHS-4 is a nationally representative large-scale sample survey that adopted a two-stage stratified random sampling design and covered 601,509 households across all states and union territories of India. The survey collected data on a range of issues, such as nutritional status, immunization, morbidity, health-seeking behavior, etc., for 259,627 children born during the five years preceding the survey to mothers aged 15–49 years. Of all these children, the height/weight measurements of 34,625 children were either missing, flagged, or out of plausible limits, because of which they were not included in the present analysis. Moreover, since the objective of the study is focused on drawing inter-group comparisons between Hindus (caste-disaggregated) and Muslims, 26,672 children belonging to households following religions other than Hinduism or Islam and 2006 Hindu children whose caste data were missing were consequently removed. Additionally, 2213 cases for which some covariates had missing values were also excluded from the study, leaving a final sample size of 194,111 children under the age of 5 years for the study.

### Outcome variables

Three indicators of malnutrition- stunting, wasting, and underweight were chosen as the outcomes in our study. Stunting measures linear growth retardation and cumulative growth deficits and indicates chronic undernourishment; wasting measures body mass relative to body height, indicating acute undernourishment; underweight indicates both acute and chronic undernutrition. Anthropometric measurements of weight and height (recumbent length for children under 24 months old and the standing height for children older than 24 months) were taken by the NFHS-4 surveyors, while the mothers or caretakers reported the age of the children. Height-for-age, weight-for-height, and weight-for-age standard deviations (z-scores) were calculated based on these measurements as per the WHO new Child Growth Standards [36], a widely accepted international standard for ‘assessing the physical growth, nutritional status and motor development in all children from birth to age five years.‘ Children whose height-for-age, weight-for-height, and weight-for-age z-scores were below minus two standard deviations (-2 SD) from the median of the reference population were considered short for their age (stunted = 1, else 0), thin (wasted = 1, else 0), and underweight (= 1, else 0), respectively.

### Independent variables

The two religious groups- Hindus and Muslims, were included in the study in light of its central objective of unraveling the determinants of Hindu-Muslim differential in the prevalence of malnutrition among under-5 children in India. However, the Hindus were further disaggregated into two sub-groups: low-caste Hindus (LCs), comprising SCs and STs, and high-caste Hindus (HCs), comprising Other Backward Classes and others, to account for the historical disadvantage and marginalization suffered by the lower caste groups in the caste system, a form of hierarchical social stratification persisting in India, which constitutes an essential axis of inequality in the Indian society [37]. While Muslims also have castes in their community, they do not share the same history as the Hindu LCs and, thus, have been retained as an aggregated group in the present study [24].

Additionally, in view of the analytical framework for the study of proximate determinants of child health that includes biological factors, maternal factors, and environmental contamination factors, among others, three broad domains of predictor variables pertaining to the child’s characteristics, mother’s characteristics and household-level factors were included in the models [38]. The child’s characteristics included age, sex, and birth order. Moreover, considering the health benefits of colostrum and early initiation of breastfeeding within an hour of birth as recommended by the WHO, ‘the time after the birth at which the child was first breastfed’ was also included in the study [39]. In the bivariate analyses, the child’s age was taken as categories (0, 1, 2, 3, and 4 years), while in the regression models, age (in months) was included as a continuous variable.

The variables related to the mother’s characteristics considered in the study were her age at the respective child’s birth, her level of education (none, incomplete primary, primary, incomplete secondary, and so on), and her Body Mass Index (categorized as underweight if below 18.5, normal if in the range 18.5–24.9 and overweight if 25 or above). Mother’s age at birth was categorized into six age groups for the bivariate analysis but taken as a continuous variable (single years of age) in the multivariate models. Besides, age-squared was also controlled in the regressions to model the effect of age more accurately, which may have a non-linear relationship with the outcomes. For example, the effect of a mother’s age could be negative initially up till, say, 30 years, and positive after that. Similarly, years of education as a continuous variable was considered for the regression analyses.

Lastly, household characteristics included the place of residence (rural/ urban), wealth index (quintiles), drinking water treatment, toilet facilities, and region (north, central, east, northeast, west, and south). The wealth index is a composite measure of a household’s cumulative living standard, calculated by principal components analysis using data on a household’s ownership of selected assets, materials used for housing construction, and types of water access and sanitation facilities. Irrespective of the source of drinking water (improved/ unimproved), we considered ‘whether anything is done to water to make it safe to drink,‘ thereby getting a binary category treated/ not treated. Toilet facilities were dichotomized as some facilities (including improved/ unimproved/ shared facilities) and no facility (in which case the household uses open spaces/fields).

The independent variables were tested for multicollinearity and the mean Variance Inflation Factor (VIF) was 1.70 indicating very low and tolerable correlation among the predictors [40].

### Statistical analyses

Descriptive statistics were calculated to understand the distribution of the sample as a whole as well as socio-religious group-wise by select background characteristics. Bivariate percentage distribution (cross-tabulation) was calculated to estimate the differentials in the prevalence of each malnutrition indicator by predictor variables. The results were tested for statistical significance using Pearson’s Chi-squared test for homogeneity or independence.

Two maximum likelihood binary logistic regression models were used to capture the crude and the adjusted association between the prevalence of malnutrition and religious/caste identity. The multivariate model on the adjusted association between under-nutrition and socio-religious affiliation controlled for all the covariates comprising the child’s, mother’s, and household’s characteristics. The results are presented as crude (COR) and adjusted odds ratios (AOR) with 95% confidence intervals (CI).

Finally, to decompose the inter-group differences (between Muslims and high-caste Hindus, as well as Muslims and low-caste Hindus) in the prevalence of stunting, wasting, and underweight among the under-5 child population in India into the significant contributing factors, Fairlie’s decomposition technique [41, 42], which is a non-linear approximation of the Blinder-Oaxaca decomposition method [43–45], was employed.

The average difference in the prevalence rates of undernutrition (stunting, wasting, and underweight) between Hindu (high-caste/ low-caste) and Muslim children may be expressed as:

$${\overline{Y}}^{H}- {\overline{Y}}^{M }= \left[\sum _{i=1}^{{N}^{H}}\frac{F\left({X}_{i}^{H}{\widehat{\beta }}^{H}\right)}{{N}^{H}}-\sum _{i=1}^{{N}^{M}}\frac{F\left({X}_{i}^{M}{\widehat{\beta }}^{M}\right)}{{N}^{M}} \right]+\left[\sum _{i=1}^{{N}^{M}}\frac{F\left({X}_{i}^{M}{\widehat{\beta }}^{H}\right)}{{N}^{M}}-\sum _{i=1}^{{N}^{M}}\frac{F\left({X}_{i}^{M}{\widehat{\beta }}^{M}\right)}{{N}^{M}} \right]$$ (Fairlie, 1999)

where,

${\overline{Y}}^{H}and {\overline{Y}}^{M }$ *are* the average probability of the binary outcome, i.e., the prevalence rate of undernutrition of Hindu (high-caste/ low-caste) and Muslim children, respectively,

*N* ^*H*^ and *N* ^*M*\^ are the sample sizes for Hindu (high-caste/ low-caste) and Muslim children, respectively,

*F* is the cumulative distribution function of logistic distribution,

${X}_{i}^{H}$ and ${X}_{i}^{M}$ are the row vectors of average values of the independent variables, and

${\widehat{\beta }}^{H}$ and ${\widehat{\beta }}^{M}$ are the vectors of coefficient estimates for Hindu (high-caste/ low-caste) and Muslim children, respectively.

The first term in brackets in the above equation represents the part of the socio-religious differential in the prevalence of undernutrition occurring due to the differences in the characteristics of the two groups, constituting the relative contribution of each of the observed predictor variables. The second term represents the degree to which Hindu (high-caste/ low-caste) and Muslim children with similar observable characteristics have different prevalence rates of undernutrition and also captures the portion of the socio-religious gap due to group differences in unmeasurable or unobserved endowments, constituting the ‘unexplained’ or residual part of the differences.

The decomposition was undertaken using the pooled estimated coefficients of the groups- Muslims and Hindus (HC/ LC). The *fairlie* command in STATA version 16 was used with the randomized ordering^1^ of the variables and 5000 decomposition replications [41]. The analyses applied the sample weights to account for the complex sample design and non-response as per the NFHS-4 (2015-16).

## Results

### Socio-religious differentials in demographic and socioeconomic characteristics of the study sample

Muslim and Hindu children constituted 16.6% and 78.7% of the under-5 child population, respectively (NFHS-4, 2015-16). The distribution of the sample of under-5 children considered in the present study is presented in Table 1. Muslim children constituted a higher proportion of birth order four or more compared to both high and low-caste Hindus by 11.5%-points and 6.2%-points, respectively. A higher share of Muslims (2.5%) was born to mothers aged 18 years or less compared to HCs (1.6%) and LCs (2.2%). The community differential also disfavoured Muslims in terms of the time after birth when first put to the breast, with only 69% of Muslim children breastfed within the first two hours after birth compared to 74% of Hindus. Besides, a higher share of Muslim children was reported to have never been breastfed than their Hindu counterparts (by 1.8%-points). While the proportion of Muslim children whose mothers received no education was much higher than HCs (by 13.5%-points), the LCs were as disadvantageous as the Muslims, with a similar proportion of children born to uneducated mothers (37.5% each). Both the low caste and high caste Hindus had a higher share of underweight mothers than Muslims by 8.5 and 1.6%-points, respectively. A higher percentage of Muslims resided in rural areas compared to Hindus. While a higher proportion of LCs belonged to the poorest wealth quintile (by 15.2%-points), a lower share of HCs belonged to the poorest quintile (by 3.5%-points) compared to Muslims. A higher percentage of Muslim households do not treat the water before drinking compared to both HCs and LCs. In contrast, a higher share of Hindu households (both caste groups) lack access to toilet facilities and resort to open defecation practices compared to Muslim households. Thus, while Muslims perform poorly in certain indicators than the high-caste Hindus, they fare better than the lower castes, justifying the disaggregation of the Hindus into the caste sub-groups for more reliable results.

**Table 1:** Religious group-wise profile of the sample (children aged below five years) by select background characteristics

Background Characteristics	Hindu	Muslim	Low caste Hindu	High caste Hindu	Total
Child's	%	%	%	%	%
**Sex**					
Male	52.0	51.9	51.0	52.6	52.0
Female	48.0	48.1	49.0	47.4	48.0
**Age (years)**					
0	18.7	17.9	19.2	18.5	18.6
1	20.3	20.8	20.3	20.4	20.4
2	19.9	20.6	19.6	20.1	20.0
3	21.1	21.1	21.2	21.0	21.1
4	20.0	19.6	19.8	20.2	20.0
**Birth order**					
First	39.0	32.6	36.2	40.6	37.9
Second	33.2	27.7	31.2	34.3	32.2
Third	15.1	17.5	16.5	14.3	15.5
Fourth or more	12.8	22.3	16.1	10.8	14.4
**Breastfed**					
within 2 hours	74.2	69.2	76.7	72.8	73.3
within 24 hours	12.0	14.6	10.7	12.7	12.5
More than 24 hours after	8.6	9.2	7.4	9.3	8.7
Never breastfed	5.2	7.0	5.2	5.2	5.5
**Mother's**					
** Age at birth**					
Below 18	1.8	2.5	2.2	1.6	1.9
18-20	15.6	15.3	17.3	14.7	15.6
21-25	46.4	42.4	44.9	47.2	45.7
26-30	25.1	24.2	24.0	25.7	24.9
31-35	8.3	11.0	8.4	8.3	8.8
Above 35	2.8	4.7	3.2	2.6	3.1
**Education**					
No education	28.9	37.5	37.5	24.0	30.4
Incomplete Primary	5.8	8.1	7.4	4.8	6.2
Primary	7.8	8.7	9.0	7.1	8.0
Incomplete Secondary	37.4	34.1	34.5	39.0	36.8
Secondary	8.9	6.5	5.6	10.8	8.5
Higher	11.3	5.2	6.1	14.3	10.2
**BMI**					
Normal	60.2	59.5	59.8	60.5	60.1
Underweight	26.0	21.9	30.4	23.5	25.3
Overweight	13.8	18.6	9.8	16.0	14.6
**Household's**					
** Residence**					
Rural	25.1	39.3	19.0	28.5	27.5
Urban	75.0	60.7	81.0	71.5	72.5
** Wealth Index**					
Poorest	26.2	22.9	38.1	19.4	25.6
Poorer	22.2	23.1	25.7	20.2	22.3
Middle	20.1	19.1	18.2	21.1	19.9
Richer	17.6	20.6	11.8	20.9	18.1
Richest	14.0	14.4	6.3	18.3	14.0
** Drinking water**					
Not Treated	68.4	73.6	72.9	65.8	69.3
Treated	31.6	26.5	27.1	34.3	30.7
** Toilet**					
Some facility	51.1	72.1	38.8	58.2	54.8
Open defecation	48.9	27.9	61.2	41.8	45.2
** Region**					
North	8.6	8.9	8.7	8.6	8.7
Central	13.2	14.0	10.6	14.8	13.4
East	15.9	11.1	16.5	15.5	15.0
Northeast	15.6	19.1	14.9	16.0	16.2
West	5.1	1.7	6.5	4.4	4.5
South	41.5	45.2	42.8	40.8	42.2
Sample size	159314	34797	61333	97981	194111

The percentages (%) are weighted

Source: Authors’ calculations from National Family Health Survey, 2015-16 (NFHS-4)

### Socio-religious differentials in the prevalence of malnutrition (stunting, wasting, and underweight)

The prevalence rates of malnutrition are presented in Table 2, which shows that while Muslims have a higher rate of stunting and lower rates of wasting and underweight compared to the Hindus as an aggregated group, the picture changes when we disaggregate the Hindus into HCs and LCs. Muslims have a lower prevalence of all three measures of malnutrition than the LCs. The prevalence of stunting and being underweight is higher among Muslims than among the HCs, while it still remains the lowest in the case of wasting when compared to both LCs and HCs.

**Table 2:** Religious group-wise prevalence of malnutrition among children under 5 years

	Hindu	Muslim	Low caste	High caste	Total
Stunting (%)	38.54	39.79	43.53	35.68	38.76
Wasting (%)	21.5	19.42	23.47	20.37	21.13
Underweight (%)	36.34	34.89	41.76	33.24	36.08

All *p*-values for chi-squared test statistic were below 0.001

Source: Authors’ calculations from National Family Health Survey, 2015-16 (NFHS-4)

Table 3 shows the socio-religious differentials in all three indicators of malnutrition by select covariates. The prevalence rates of each group by covariates are shown in Appendix 1. On average, Muslim children have a lower rate of stunting than lower-caste Hindus and a higher rate of stunting than high-caste Hindus. This trend is uniform across all covariates barring a few exceptions. Muslim children with third or higher birth order and those born to mothers with no or below the secondary level of education had a marginally lower stunting rate than both caste groups of Hindus with similar attributes. Muslims belonging to poorer or middle-wealth quintiles or households with no toilet facilities had a higher rate of stunting than both HCs and LCs belonging to such households.

**Table 3:** Socio-religious differentials in the prevalence of malnutrition among children under 5 years by select covariates

Covariates	Stunting		Wasting		Underweight	
	M-LC^a^	M-HC^a^	M-LC^a^	M-HC^a^	M-LC^a^	M-HC^a^
Child's						
**Sex**						
Male	-4.0	4.6	-4.1	-0.6	-7.1	2.2
Female	-3.5	3.5	-4.0	-1.3	-6.7	1.0
**Age (years)**						
0	-2.8	1.7	-4.0	-1.6	-6.2	0.5
1	-5.5	3.6	-4.2	0.8	-8.0	2.9
2	-5.8	3.8	-4.6	-1.5	-9.3	0.8
3	-3.0	5.5	-3.8	-1.2	-5.6	2.2
4	-3.0	5.2	-3.1	-1.1	-5.9	1.6
**Birth order**						
First	-4.0	3.8	-3.6	-0.7	-6.9	1.2
Second	-4.5	3.1	-4.4	-1.5	-8.6	0.4
Third	-5.8	-0.3	-3.9	-0.9	-7.4	-1.1
Fourth or more	-4.8	-0.6	-5.0	-1.8	-7.7	-2.4
**Breastfed**						
within 2 hours	-4.1	3.6	-4.1	-1.3	-7.6	0.8
within 24 hours	-2.6	5.5	-3.1	-0.9	-4.6	3.5
More than 24 hours after	-4.2	4.8	-3.9	2.8	-7.4	4.3
Never breastfed	-3.3	4.7	-3.5	-1.4	-3.2	3.4
**Mother's**						
** Age at birth**						
Below 18	-3.5	2.0	-1.0	-0.9	-4.3	-1.2
18-20	-3.9	2.3	-5.8	-1.8	-8.3	-1.7
21-25	-4.3	3.1	-4.0	-1.2	-7.7	0.8
26-30	-3.5	4.9	-3.4	-0.5	-5.7	3.1
31-35	-4.0	5.5	-4.1	-0.6	-7.2	2.9
Above 35	-4.0	7.7	-4.0	0.9	-5.3	7.1
** Education**						
No education	-2.6	-0.5	-5.4	-1.8	-6.5	-2.3
Incomplete primary	1.2	2.8	-5.0	-2.7	-5.0	-2.1
Primary	-6.1	-1.7	-3.0	-2.8	-7.4	-3.3
Incomplete secondary	-6.0	-0.2	-2.8	-0.8	-7.5	-2.1
Secondary	-3.2	0.9	-5.3	-1.8	-9.2	-1.4
Higher	-3.8	0.3	-2.0	0.2	-7.0	-2.2
** BMI**						
Normal	-2.4	4.4	-2.2	-0.4	-4.0	2.3
Underweight	-2.8	3.1	-6.2	-2.0	-8.0	0.0
Overweight	-1.2	6.3	-0.5	-0.3	-2.5	4.2
'**Household's**						
** Residence**						
Rural	-2.3	5.9	-1.2	0.2	-2.7	4.3
Urban	-1.8	4.8	-4.9	-1.5	-6.4	1.5
** Wealth Index**						
Poorest	-0.4	0.8	-5.7	-1.9	-6.1	-2.0
Poorer	0.8	1.9	-4.1	-3.2	-3.4	-1.8
Middle	2.4	5.6	-2.5	-0.1	0.3	3.2
Richer	-1.4	2.9	-1.9	-0.3	-1.9	1.9
Richest	-2.1	3.3	2.2	0.4	-2.7	1.9
** Drinking water**						
Not Treated	-1.7	4.2	-3.8	-0.5	-5.1	2.0
Treated	-9.5	0.8	-4.7	-1.6	-11.9	-1.5
** Toilet**						
Some facility	1.0	7.0	-2.4	-0.5	-1.7	4.0
Open defecation	1.0	4.8	-3.8	-0.8	-3.1	2.6
** Region**						
North	3.7	7.3	-1.5	0.1	-0.8	3.5
Central	-6.2	3.4	-1.7	1.5	-6.7	2.6
East	-11.8	0.0	-6.2	-1.9	-14.1	-2.8
Northeast	-8.1	-0.5	-6.9	-2.8	-13.7	-3.9
West	-7.8	7.2	-9.8	-4.6	-14.6	0.5
South	-1.0	6.1	-3.0	-0.5	-3.1	4.7
Total	-3.7	4.1	-4.1	-0.95	-6.9	1.7

*M *Muslims, *HC *High-caste Hindus, *LC *Low-caste Hindu

^a^percentage-point differences

In the case of wasting, Muslims, on average, had the lowest rate of prevalence. This trend was consistent in all covariates except for a few like children aged one year, breastfed more than 24 h after birth, born to mothers with higher education, residing in rural India, north and central region, in which case, Muslims had a higher rate of wasting than high-caste Hindus but lower rate than LCs. However, among the children born to the wealthiest households, Muslims had the highest rate of wasting compared to both caste groups of Hindus.

Finally, in the case of the prevalence of underweight, Muslims had an advantage over low-caste Hindus and a disadvantage compared to high-caste Hindus, on average. This pattern remained consistent for all covariates, albeit with certain exceptions. The high-caste Hindu advantage over Muslims reversed in favor of Muslims in the case of children born in birth order of three or more, born to mothers below the age of 20 years, belonging to poorest/ poorer households, those with access to safe drinking water, and residing in the east and northeast regions of India. Also, Muslim children exhibited an advantage over both LCs and HCs in the prevalence of underweight across all categories of mothers’ education. This differential, however, was the widest in the case of primary education and lowest for the secondary level of education.

All *p*-values for chi-squared test statistic were below 0.05.

Source: Authors’ calculations from National Family Health Survey, 2015-16 (NFHS-4).

### Association between socio-religious affiliation and prevalence of malnutrition

The crude and adjusted odds ratios computed through logistic regression to assess the effect of socio-religious affiliation on the prevalence of malnutrition have been presented in Table 4. The results of the crude analysis indicated that the odds of stunting are 17% higher among LCs and 16% lower among HCs with reference to Muslim children. However, both caste groups of Hindus have a higher likelihood of wasting than Muslims, by 16% and 6%, respectively. After controlling for the effect of a vector of covariates, the direction of socio-religious differentials remained the same, although the magnitude shrunk in both stunting and wasting. In the case of underweight, LCs have 34% higher odds, while HCs have 7% lower odds with respect to Muslims. In the adjusted model, however, the high-caste Hindu advantage over Muslims is reversed, and HCs show a controlling 4% higher odds of being underweight than Muslims.

**Table 4:** Association between socio-religious affiliation and malnutrition among children under 5 years

Outcome variable	Stunting		Wasting		Underweight	
Covariates	COR	AOR	COR	AOR	COR	AOR
**Religion/Caste**						
** Muslims®**						
Low-caste Hindu	1.17*** (1.12-1.21)	1.05** (1.01-1.10)	1.27*** (1.21-1.34)	1.16*** (1.10-1.22)	1.34*** (1.29-1.39)	1.16*** (1.12-1.22)
High-caste Hindu	0.84*** (0.81-0.87)	0.96** (0.92-1.00)	1.061** (1.01-1.11)	1.06** (1.01-1.12)	0.93*** (0.90-0.96)	1.04* (0.99-1.08)
** Child's Age**						
Child's age (months)		1.64*** (1.60-1.69)		0.74*** (0.71-0.76)		1.18*** (1.15-1.22)
Child's age-squared		0.99*** (0.99-1.00)		1.00*** (1.00-1.00)		0.99*** (0.99-1.00)
** Sex**						
Male®						
Female		0.94*** (0.92-0.97)		0.89*** (0.86-0.92)		0.95*** (0.93-0.98)
** Birth-order**						
First®						
Second		1.16*** (1.12-1.20)		0.99* (0.95-1.03)		1.12*** (1.08-1.16)
Third		1.25*** (1.20-1.31)		0.99 (0.94-1.05)		1.19*** (1.14-1.24)
Fourth or more		1.46*** (1.38-1.53)		1.01 (0.95-1.08)		1.36*** (1.29-1.43)
** Breastfed**						
within 2 hours®						
within 24 hours		1.00 (0.96-1.04)		0.92** (0.88-0.96)		1.01 (0.98-1.06)
More than 24 hours after		1.12***(1.07-1.17)		1.01 (0.95-1.07)		1.13*** (1.08-1.19)
Never breastfed		1.02 (0.96-1.08)		0.9** (0.84-0.97)		0.95(0.89-1.010)
** Mother's age**						
Mother's age at birth (years)		0.94*** (0.92-0.96)		0.99 (0.97-1.02)		0.95*** (0.93-0.97)
Age at birth squared		1.00*** (1.00-1.00)		1.00 (1.00-1.00)		1.00** (1.00-1.00)
** Mother's Education**						
Years of education		0.960*** (0.957-0.964)		0.99*** (0.99-1.00)		0.96*** (0.96-0.97)
** Mother's BMI**						
Normal®						
Underweight		1.21*** (1.17-1.24)		1.35*** (1.31-1.40)		1.56*** (1.52-1.61)
Overweight		0.79*** (0.75-0.82)		0.70*** (0.66-0.74)		0.68*** (0.65-0.72)
** Residence**						
Urban®						
Rural		0.93*** (0.90-0.97)		0.90*** (0.86-0.95)		0.87*** (0.84-0.91)
** Wealth Index**						
Poorest®						
Poorer		0.89*** (0.86-0.92)		0.90*** (0.87-0.94)		0.87*** (0.84-0.9)
Middle		0.78*** (0.74-0.81)		0.84*** (0.80-0.89)		0.74*** (0.71-0.77)
Richer		0.66*** (0.62-0.69)		0.84*** (0.79-0.9)		0.65*** (0.62-0.69)
Richest		0.56*** (0.52-0.60)		0.80*** (0.73-0.86)		0.53*** (0.49-0.57)
** Drinking water**						
Not treated®						
Treated		0.92*** (0.89-0.95)		1.10*** (1.06-1.14)		0.96** (0.93-0.99)
** Toilet Facility**						
Some facility®						
Open Defecation		1.16*** (1.12-1.2)		1.06** (1.02-1.11)		1.14*** (1.10-1.18)
** Region**						
North®						
Central		1.21*** (1.14-1.27)		1.03 (0.97-1.10)		1.10** (1.04-1.16)
East		1.14*** (1.08-1.21)		1.43*** (1.34-1.53)		1.28*** (1.21-1.36)
Northeast		1.20*** (1.14-1.27)		1.38*** (1.30-1.47)		1.2*** (1.14-1.27)
West		0.90** (0.83-0.98)		1.01 (0.93-1.09)		0.92* (0.85-1.00)
South		1.13*** (1.08-1.18)		1.04 (0.98-1.10)		1.01 (0.97-1.07)
Wald Statistic	532.03	7278.78	150.08	2443.97	674.96	7007.72
Degrees of freedom	2	28	2	28	2	28
Prob > chi2	<0.001	<0.001	<0.001	<0.001	<0.001	<0.001
Pseudo R2	0.0038	0.0591	0.0014	0.0242	0.0047	0.0569
No. of observations	194111	194111	194111	194111	194111	194111

**®**Reference category; *** *p* < 0.001 ** *p* < 0.05 and * *p* < 0.1

Source: Authors’ calculations from National Family Health Survey, 2015-16 (NFHS-4)

The results of the adjusted models further indicate a monotonic increasing function of stunting and underweight by child’s age until a turning point is reached, after which the function starts to decrease. Female children have lower odds of malnutrition than males in the case of all three indicators. The odds of undernutrition increase with increasing birth order and vice versa. Also, the earlier the child is put to the breast after birth, the lower the odds of malnourishment. The mother’s age at the child’s birth is a significant determinant only in stunting and underweight. The odds of undernutrition decrease with the increasing age of the mother up until a point post which the chances of undernutrition are higher with increasing mother’s age. There is a monotonic decreasing function of malnutrition by the mother’s years of education. Children of underweight mothers are more likely to be malnourished compared to mothers with BMI in the normal range. Rural children have lower odds of undernutrition compared to their urban counterparts. The odds of malnourishment keep declining as we move up along the wealth index gradient. Children belonging to households that do not treat the water to make it safe to drink and practice open defecation have higher odds of malnutrition than those with access to safe drinking water and toilet facilities. Children residing in the western region have the lowest odds of stunting and being underweight. In contrast, those residing in the eastern region have the highest odds of wasting and being underweight among all other regions.

### Major contributors to the Muslim-Hindu (HC/LC) gap in the prevalence of stunting, wasting, and underweight

The results of the decomposition analysis, presented in Table 5, shed light on the relative contribution of the socio-religious differential in each covariate to the Muslim-Hindu (HC/LC) gap in the prevalence of malnutrition among the under-5 children in India. The set of predictors considered in the model explains roughly 79% of the high-caste Hindu advantage over Muslims in stunting prevalence. The differences in birth order, mother’s level of education, and wealth index are the major contributors, accounting for 22.4%, 51%, and 14.5% of the differences, respectively. However, in the case of the prevalence of wasting that is lower among Muslim children by roughly 1%-points compared to HCs, the differences in the select covariates suggest it should have been marginally higher than HCs by 0.4%-points, given the advantage of HCs in mother’s education and birth order. In the case of underweight, the decomposition results indicate that the HCs should have had a marginally higher advantage (2.8%-points) instead of the current advantage of 1.7%-points. The differentials in birth order (41.1%) and economic status (43%) contributed to expanding the Muslim disadvantage over high-caste Hindus in underweight. Better access to sanitation among Muslim children than among high-caste Hindus resulted in offsetting a part (27%) of their nutritional disadvantage in being underweight.

**Table 5:** Decomposition of the socio-religious differential in malnutrition among children under-5 years

	High-caste Hindus vs. Muslims						Low-caste Hindus vs. Muslims					
	Stunting		Wasting		Underweight		Stunting		Wasting		Underweight	
High/ Low Caste Hindu	0.3568		0.2037		0.3324		0.4353		0.2347		0.4176	
Muslim	0.3979		0.1942		0.3489		0.3979		0.1942		0.3489	
Difference	-0.0411		0.0095		-0.0165		0.0374		0.0406		0.0687	
Explained	-0.032		-0.0035		-0.0284		0.0258		0.0143		0.032	
	**Coefficient**	**%**	**Coefficient**	**%**	**Coefficient**	**%**	**Coefficient**	**%**	**Coefficient**	**%**	**Coefficient**	**%**
Age	-0.0003*	0.74	0.0004*	3.86	-0.0003*	2.05	0.0003*	0.85	0.0001	0.36	-0.0003*	-0.43
Sex	0.0001*	-0.24	-0.00004	-0.43	0.0001*	-0.48	-0.0001*	-0.39	-0.0003*	-0.62	-0.0001*	-0.16
Birth Order	-0.0092*	22.36	0.0001	1.13	-0.0068*	41.14	-0.0045*	-11.93	0.0002	0.4	-0.0035*	-5.05
Breastfeeding	-0.0003*	0.8	0.0003*	3.13	-0.0001	0.72	-0.0005*	-1.43	0.0006*	1.57	-0.0001	-0.19
Mother’s age at birth	0.0027*	-6.67	-0.0001	-1.25	0.0021*	-12.62	0.0027*	7.10	-0.0002	-0.37	0.0025*	3.68
Mother’s education	-0.0209*	50.89	-0.0037*	-38.96	-0.0191*	115.69	-0.0004*	-1.15	0.0001	0.22	-0.0007*	-1.00
Mother’s BMI	0.0002*	-0.57	0.0002*	2.18	0.00005	-0.28	0.0006*	1.72	0.0005*	1.15	0.0001	0.18
Residence	-0.0012*	2.91	-0.0016*	-17.25	-0.0023*	14.23	-0.0024*	-6.44	-0.0026*	-6.48	-0.0043*	-6.23
Economic Status	-0.0059*	14.47	-0.0012*	-12.81	-0.0071*	43.02	0.0212*	56.83	0.0087*	21.37	0.0255*	37.14
Drinking Water	-0.0016*	3.78	0.0009*	9.68	-0.0007*	4.04	-0.0003*	-0.70	0.0001	0.16	-0.0001*	-0.12
Toilet	0.0052*	-12.54	0.0016*	16.65	0.0045*	-27.02	0.0122*	32.69	0.0053*	13.03	0.0125*	18.19
Region	-0.0011*	2.65	-0.0003	-3.18	0.0012*	-7.51	-0.003*	-8.01	0.0016*	3.85	0.0006	0.83
Explained	-0.0323	78.59	-0.0035	-37.26	-0.0285	172.98	0.0258	69.13	0.0141	34.65	0.0322	46.84
Unexplained	-0.0088	21.41	0.0130	137.26	0.0120	-72.98	0.0115	30.87	0.0265	65.35	0.0365	53.16

* *p* < 0.05

Source: Authors’ calculations from National Family Health Survey, 2015-16 (NFHS-4)

Compared to the low-caste Hindus, Muslims consistently have an advantage in all three indicators of malnutrition considered in this study. While our model explains 69% of the differences in stunting prevalence, the majority of the Muslim advantage over LCs is explained by the socio-religious differences in wealth index (57%) and access to toilet facilities (33%). However, a part of this advantage gets offset due to the Muslim disadvantage in birth order (12%) and regional factors (8%). Moreover, less than 50% of the Muslim advantage is explained by the chosen set of variables in the case of wasting and being underweight. This implies that Muslims are favored by some unobservable attributes that work to the advantage of their nutritional status in the under-5 age group. In the case of both wasting and underweight, better economic status and access to sanitation translated into better nutritional outcomes for Muslim children in comparison to their low-caste Hindu counterparts.

### Discussion and conclusion

This paper attempted to contribute to the scarce body of research dedicated to investigating the inequalities in child nutrition status along the axis of socio-religious affiliation by quantifying the socio-religious differential in indicators of child undernutrition and decomposing the same to shed light on the major contributory factors of these differences. The study produced several interesting findings. We found the Muslims and lower-caste Hindus to be equally at a disadvantage in terms of socioeconomic status compared to the high-caste Hindus. This disadvantage exists probably because when Islam made its way to the subcontinent, it was mainly the low-caste Hindus who took refuge in Islam through religious conversions to circumvent the oppressions by the higher-caste Hindus [46]. This could, therefore, be the reason for persisting low socioeconomic conditions among Indian Muslims. The caste system, which continues to play a significant role in the country’s social and political interactions, has forced many people who belong to the lower castes into poverty [47]. To date, most of those still trapped in poverty are Dalits and tribes, especially women [48]. Dalits or ‘untouchables’ have the most menial jobs, no assets, poor education levels, and experience restricted occupational mobility [49]. The historical disadvantage of the lower caste Hindus in terms of socioeconomic status (a legacy, the traces of which is still suffered by the converted Muslims) was found to have penetrated to under-5 nutritional outcomes. Corroborating the findings of previous studies, our results indicate that Muslim and lower-caste Hindu children have a higher prevalence of stunting and being underweight than their high-caste Hindu counterparts [50]. In the case of wasting, however, Muslim children had the lowest prevalence rate, a paradox that we attempted to find an answer to at the later stage of the analysis.

The findings of this study build on existing work that illustrates the need to address socioeconomic factors to improve health outcomes. The regression analysis showed that maternal characteristics such as BMI, age at birth of the child, education, and initiation of early breastfeeding are significantly associated with nutrition outcomes of children, echoed by the findings of several other studies [51–55]. Early initiation of breastfeeding protects against diarrhea-related morbidity, reduces hospitalization episodes, and has a significant association with nutritional status [56]. But, early initiation of breastfeeding is less common among Muslims than Hindus, as highlighted in the study. It was revealed in the decomposition results that this disadvantage in breastfeeding practices among Muslims contributed to offsetting their nutritional advantage over low-caste Hindus. Moreover, educated mothers possess improved information acquisition skills, positive caring behavior, follow dietary recommendations, interact with health professionals effectively, and improve the intrahousehold allocation of resources in favor of the children [52–55]. The ramifications of the intergenerational cycle of malnutrition are well documented. Mothers who have lower BMI have higher odds of stunted children [57]. The lower the mother’s weight, the higher the risk of infants being born underweight due to intrauterine growth rate reduction[58]. The odds of stunting tend to be lower among children whose mother’s age at birth falls in the age group of 20–34 years than those below 20 years of age [51, 59]. However, Muslim mothers fare worse than high caste Hindus in the case of each of these maternal characteristics, which have a significant association with the nutrition outcomes of the children, the effect of which was seen to be expanding the Muslim disadvantage in stunting and underweight than high-caste Hindus in the decomposition results. The relation of higher birth order with poor nutritional status is also well-established. Postnatal care for children of higher birth order is neglected as the intrahousehold resource allocation decreases with increasing birth order [60, 61]. In our study, Muslims were found to have a higher birth order (of 4 or more) than both high-caste and low-caste Hindus. Resultantly, birth order acted in favor of both caste-disaggregated groups of Hindus in the decomposition. Regarding household-level factors, access to sanitation has a vital role in determining the prevalence of undernutrition with higher odds of stunting among the children who practice open defecation than the users of improved toilet facilities [62]. Compared to Hindu households, a lesser proportion of Muslim households practice open defecation, translating into Muslim advantage in nutrition status over Hindus [21].

The relatively poor socioeconomic characteristics of Muslims compared to high-caste Hindus and, at times, even lower-caste Hindus should put Muslims at a nutritional disadvantage. However, their nutritional advantage over high-caste Hindus in wasting is a paradox. While the poor performance of Muslim children compared to high-caste Hindus, in stunting and underweight, is explained mainly by the chosen set of variables in our model, their comparatively better performance in wasting remained a puzzle, nonetheless. The cultural factors such as washing hands before prayers which improves personal hygiene; closely-knit social networks and kinship structures expressed by endogamy; and lower prevalence of dowry and son preference might reflect a comparatively higher status accorded to women in Muslim households [24, 28]. This suggests that the Muslim advantage over high-caste Hindus in wasting may have been rendered by behavioral and cultural differences and can be explained by the Particularized Theology Hypothesis. On the flip side, the Muslim advantage over lower-caste Hindus could be partially ascertained by the former’s better economic status than lower-caste Hindus, as highlighted by the decomposition analysis results. The economic status of the households has a significant association with undernutrition, as wealthier households are known to have a lesser prevalence of undernutrition than poorer households [63, 64]. Muslim households were found to be poorer than upper-caste Hindus but better off than Hindu lower castes on average, as also noted in the post-Sachar evaluation report. The differential in the prevalence of undernutrition between Muslims and low-caste Hindus is, thus, better explained by the Selectivity Hypothesis.

Studies show discrimination against SC/ST women and children in access to food security-related services- mid-day meal scheme, ICDS services, public distribution system, primary health services, etc., which adversely affects their nutrition status [65–67]. This calls for the implementation of instruments designed to address the child malnutrition crisis in India through efficient targeting of beneficiaries. While the budgetary allocation to the various existing schemes, such as ICDS, POSHAN Abhiyan, etc., needs to be significantly increased, the shortfalls in implementation also need to be improved. Audit reports have revealed deficits in the disbursal of funds as well as actual spending of disbursed funds compared to the budgetary allocations made for child nutrition schemes [68]. Malnourished children are nine times more likely to die than healthy children [69]. There are significant direct and indirect economic losses due to undernutrition. Direct productivity losses have been estimated at more than 10% of lifetime personal income and roughly 3% loss to the Gross Domestic Product in India [70]. Besides, adverse health shocks during early childhood have been proven to cast long-lasting adverse spill-over effects well into adulthood, leading to poor health stock and increased healthcare costs, constituting indirect losses [71].

Our study reinforces the findings from the previous studies that the position of Muslims, SCs, and STs is noticeably more vulnerable than that of high-caste Hindus. Equitable access to education, poverty alleviation and better employment opportunities also need policy attention as they have demonstrated a strong bearing on child nutrition outcomes. Although levels of income, education, and access to public health care are all strongly correlated with nutritional status, social membership also plays a role in exacerbating nutritional inequality [72]. These findings have two distinct policy implications. They demand targeted policy measures that specifically protect against the marginalization of the SC, ST, and Muslim populations in addition to general policy measures that are common to all socio-economically poorer sections of the society (irrespective of the socio-religious affiliation). Uplifting the socioeconomic status of the poor through redistributive mechanisms ensuring greater access to assets and wages, which is necessary for better diet and access to healthcare, is another important policy implication of this study. Besides, in the case of the SC and ST, who often experience discrimination when trying to access sources of livelihood, education, public health services, food security, etc., in addition to these common measures, supplemental policy measures are imperative to circumvent the barriers imposed by social exclusion and marginalization.

The present study was not without limitations. In the absence of a multilevel analytical model, the variation in nutrition outcomes attributable to different population levels, such as neighborhoods, wards, etc., within which individuals are nested, could not be captured in our single-level analysis. Also, the cross-sectional design of the study did not allow for making definitive inferences about the direction of cause and effect. However, despite these limitations, the study made a modest yet integral contribution to the understanding of the determinants of socio-religious differences in the prevalence of undernutrition among children below five years of age in India, drawing evidence from the most recent nationally representative sample survey dataset available.

### Supplementary Information

**Additional file 1: Appendix 1.** Religious group-wise prevalence of malnutrition among children under-5 years, by select covariates.

## Data Availability

The study is based on the National Family Health Survey- 4 (2015-16), a secondary source of de-identified data available in the public domain upon request from the Demographic and Health Surveys (DHS) Program. The data can be accessed from: https://dhsprogram.com/methodology/survey/survey-display-355.cfm.

## Abbreviations

AOR: Adjusted Odds Ratio
COR: Crude Odds Ratio
HCs: High-Caste Hindus
LCs: Low-Caste Hindus
NFHS: National Family Health Survey
SCs: Scheduled Castes
STs: Scheduled Tribes
VIF: Variance Inflation Factor
WHO: World Health Organisation

## Footnotes

1 In order to address the concern that the decomposition estimates could be sensitive to the ordering of variables because of the nonlinearity of the prediction equations (Fairlie, 2017), the *‘ro’* option was added to the *‘fairlie’* command in STATA to randomize the order of the variables in each replication and, return the approximate average results over all possible orderings [44].
