# Supplementary material for: Exploring the paradox of Muslim advantage in undernutrition among under-5 children in India: a decomposition analysis
Source: BMC Pediatr. 2023 Oct 16;23:515. doi: 10.1186/s12887-023-04345-y (PMC10578034; doi:10.1186/s12887-023-04345-y)
Supplement: Supplementary file 1 — Additional file 1: Appendix 1. Religious group-wise prevalence of malnutrition among children under-5 years, by select covariates. [file 12887_2023_4345_MOESM1_ESM.docx]

# Appendix

Appendix 1. Religious group-wise prevalence of malnutrition among children under-5 years, by select covariates

| Covariates | | STUNTING | | | | WASTING | | | | UNDERWEIGHT | | | |
| --- | --- | --- | --- | --- | --- | --- | --- | --- | --- | --- | --- | --- | --- |
|  |  | Muslim | Low caste Hindu | High caste Hindu | Total | Muslim | Low caste Hindu | High caste Hindu | Total | Muslim | Low caste Hindu | High caste Hindu | Total |
| Child’s |  | % | % | % | % | % | % | % | % | % | % | % | % |
| Sex | Male | 40.5 | 44.5 | 35.9 | 39.2 | 20.5 | 24.6 | 21.1 | 22.0 | 35.5 | 42.6 | 33.3 | 36.4 |
|  | Female | 39.0 | 42.5 | 35.5 | 38.3 | 18.3 | 22.3 | 19.6 | 20.2 | 34.2 | 40.9 | 33.2 | 35.7 |
| Age (years) | 0 | 21.8 | 24.6 | 20.1 | 21.8 | 27.4 | 31.4 | 29.0 | 29.5 | 26.3 | 32.5 | 25.8 | 28 |
|  | 1 | 43.4 | 48.9 | 39.8 | 43.1 | 21.4 | 25.6 | 20.6 | 22.2 | 34.7 | 42.7 | 31.8 | 35.6 |
|  | 2 | 43.5 | 49.3 | 39.7 | 43.2 | 16.9 | 21.5 | 18.4 | 19.0 | 35.7 | 45 | 34.9 | 38 |
|  | 3 | 45.7 | 48.7 | 40.2 | 43.7 | 16.1 | 19.9 | 17.3 | 17.8 | 38.2 | 43.8 | 36 | 38.8 |
|  | 4 | 42.2 | 45.2 | 37.0 | 40.4 | 16.3 | 19.4 | 17.4 | 17.8 | 38.5 | 44.4 | 36.9 | 39.4 |
| Birth order | First | 34.7 | 38.7 | 30.9 | 33.7 | 19.2 | 22.8 | 19.9 | 20.6 | 30.6 | 37.5 | 29.4 | 31.9 |
|  | Second | 37.8 | 42.3 | 34.7 | 37.4 | 18.8 | 23.2 | 20.3 | 20.9 | 32.5 | 41.1 | 32.1 | 34.7 |
|  | Third | 41.1 | 46.9 | 41.4 | 43.1 | 19.9 | 23.8 | 20.8 | 21.6 | 36.7 | 44.1 | 37.8 | 39.6 |
|  | Fourth or more | 48.7 | 53.5 | 49.3 | 50.5 | 20.1 | 25.1 | 21.9 | 22.5 | 42.7 | 50.4 | 45.1 | 46.2 |
| Breastfed | within 2 hours | 39.0 | 43.1 | 35.4 | 38.4 | 19.7 | 23.8 | 21.0 | 21.6 | 34 | 41.6 | 33.2 | 36 |
|  | within 24 hours | 40.2 | 42.8 | 34.7 | 37.9 | 18.1 | 21.2 | 19.0 | 19.4 | 35.7 | 40.3 | 32.2 | 35 |
|  | More than 24 hours after | 43.2 | 47.4 | 38.4 | 41.6 | 21.9 | 25.8 | 19.1 | 21.3 | 39.1 | 46.5 | 34.8 | 38.6 |
|  | Never breastfed | 42.4 | 45.7 | 37.7 | 41.0 | 16.5 | 20.0 | 17.9 | 18.2 | 36.8 | 40 | 33.4 | 36 |
| Mother’s |  |  |  |  |  |  |  |  |  |  |  |  |  |
| Age at birth | Below 18 | 40.7 | 44.2 | 38.7 | 41.0 | 19.0 | 20.0 | 19.9 | 19.7 | 37 | 41.3 | 38.2 | 39 |
|  | 18-20 | 40.2 | 44.1 | 37.9 | 40.3 | 18.1 | 23.9 | 19.9 | 20.9 | 33.7 | 42 | 35.4 | 37.3 |
|  | 21-25 | 38.3 | 42.6 | 35.2 | 37.9 | 19.4 | 23.4 | 20.6 | 21.2 | 33.8 | 41.5 | 33 | 35.6 |
|  | 26-30 | 39.3 | 42.8 | 34.4 | 37.7 | 19.7 | 23.1 | 20.2 | 20.9 | 34.8 | 40.5 | 31.7 | 34.8 |
|  | 31-35 | 42.0 | 46.0 | 36.5 | 40.4 | 20.0 | 24.1 | 20.6 | 21.4 | 36.4 | 43.6 | 33.5 | 37 |
|  | Above 35 | 48.7 | 52.7 | 41.0 | 46.6 | 21.4 | 25.4 | 20.5 | 22.2 | 44.3 | 49.6 | 37.2 | 42.9 |
| Education | No education | 49.7 | 52.3 | 50.2 | 50.9 | 20.0 | 25.4 | 21.8 | 22.8 | 43.6 | 50.1 | 45.9 | 47 |
|  | Incomplete primary | 46.7 | 45.5 | 43.9 | 45.1 | 19.3 | 24.3 | 22.0 | 22.2 | 39.5 | 44.5 | 41.6 | 42.2 |
|  | Primary | 39.4 | 45.5 | 41.1 | 42.3 | 18.4 | 21.4 | 21.2 | 20.7 | 34.9 | 42.3 | 38.2 | 38.9 |
|  | Incomplete secondary | 32.5 | 38.5 | 32.7 | 34.3 | 19.7 | 22.5 | 20.5 | 20.9 | 29.3 | 36.8 | 31.4 | 32.6 |
|  | Secondary | 27.6 | 30.8 | 26.7 | 27.7 | 17.2 | 22.5 | 19.0 | 19.5 | 22.8 | 32 | 24.2 | 25.6 |
|  | Higher | 20.8 | 24.6 | 20.5 | 21.3 | 17.9 | 19.9 | 17.7 | 18.1 | 16.4 | 23.4 | 18.6 | 19.3 |
| BMI | Normal | 40.1 | 42.5 | 35.7 | 38.5 | 19.6 | 21.8 | 20.0 | 20.5 | 34.6 | 38.6 | 32.3 | 34.6 |
|  | Underweight | 46.4 | 49.2 | 43.3 | 45.9 | 23.5 | 29.7 | 25.5 | 26.7 | 45 | 53 | 45 | 47.9 |
|  | Overweight | 30.9 | 32.1 | 24.6 | 27.5 | 14.0 | 14.5 | 14.3 | 14.3 | 23.9 | 26.4 | 19.7 | 22 |
| Household’s |  |  |  |  |  |  |  |  |  |  |  |  |  |
| Residence | Rural | 34.2 | 36.5 | 28.3 | 31.5 | 20.0 | 21.2 | 19.8 | 20.1 | 31.4 | 34.1 | 27.1 | 29.6 |
|  | Urban | 43.4 | 45.2 | 38.6 | 41.5 | 19.1 | 24.0 | 20.6 | 21.5 | 37.2 | 43.6 | 35.7 | 38.6 |
| Wealth Index | Poorest | 51.7 | 52.1 | 50.9 | 51.6 | 20.9 | 26.6 | 22.8 | 24.2 | 45.2 | 51.3 | 47.2 | 48.7 |
|  | Poorer | 44.8 | 44.0 | 42.9 | 43.6 | 18.9 | 23.0 | 22.1 | 21.8 | 38.6 | 42.0 | 40.4 | 40.6 |
|  | Middle | 40.4 | 38.0 | 34.8 | 36.6 | 19.4 | 21.9 | 19.5 | 20.1 | 35.2 | 34.9 | 32 | 33.4 |
|  | Richer | 31.0 | 32.4 | 28.1 | 29.5 | 18.9 | 20.8 | 19.2 | 19.5 | 28.5 | 30.4 | 26.6 | 27.7 |
|  | Richest | 24.7 | 26.8 | 21.4 | 22.7 | 18.7 | 16.5 | 18.3 | 18.1 | 21.5 | 24.2 | 19.6 | 30.6 |
| Drinking water | Not Treated | 43.3 | 45.0 | 39.1 | 41.7 | 19.2 | 23.0 | 19.7 | 20.6 | 37.6 | 42.7 | 35.6 | 38.2 |
|  | Treated | 30.0 | 39.5 | 29.2 | 32.0 | 20.1 | 24.8 | 21.7 | 22.2 | 27.3 | 39.2 | 28.8 | 31.3 |
| Toilet | Some facility | 35.9 | 34.9 | 28.9 | 31.8 | 18.7 | 21.1 | 19.2 | 19.5 | 31.2 | 32.9 | 27.2 | 29.3 |
|  | Open defecation | 50.0 | 49.0 | 45.2 | 47.2 | 21.2 | 25.0 | 22.0 | 23.1 | 44.3 | 47.4 | 41.7 | 44.3 |
| Region | North | 39.6 | 35.9 | 32.3 | 34.7 | 18.5 | 20.0 | 18.4 | 18.9 | 34.6 | 35.4 | 31.1 | 33.0 |
|  | Central | 48.8 | 55.0 | 45.4 | 48.3 | 21.4 | 23.1 | 19.9 | 20.9 | 43.8 | 50.5 | 41.2 | 43.9 |
|  | East | 33.6 | 45.4 | 33.6 | 37.5 | 22.7 | 28.9 | 24.6 | 25.8 | 32.8 | 46.9 | 35.6 | 39.0 |
|  | Northeast | 33.4 | 41.5 | 33.9 | 35.9 | 20.2 | 27.1 | 23.0 | 23.6 | 28.4 | 42.1 | 32.3 | 34.2 |
|  | West | 33.8 | 41.6 | 26.6 | 33.5 | 12.8 | 22.6 | 17.4 | 19.3 | 26.4 | 41.0 | 25.9 | 32.4 |
|  | South | 41.5 | 42.5 | 35.4 | 38.7 | 18.1 | 21.1 | 18.6 | 19.3 | 35.8 | 38.9 | 31.1 | 34.4 |
| Total | | 39.8 | 43.5 | 35.7 | 38.8 | 19.4 | 23.5 | 20.4 | 21.1 | 34.9 | 41.8 | 33.2 | 36.1 |

Note: all p-values for chi squared test statistic were below 0.05, indicating statistical significance of the bivariate relationships at 95% CI

Source: Authors’ own calculations from National Family Health Survey, 2015-16 (NFHS-4)
